# The Management of Xylazine Overdose With Naloxone

**DOI:** 10.7759/cureus.57638

**Published:** 2024-04-05

**Authors:** James Morris, Dustin Hoang

**Affiliations:** 1 Emergency Medicine, Texas Tech University Health Sciences Center School of Medicine, Lubbock, USA

**Keywords:** emergency department, horse tranquilizer, naloxone, xylazine, toxicology

## Abstract

This article discusses a rare case of isolated xylazine overdose in a human, treated successfully with naloxone. Xylazine, typically used as a veterinary tranquilizer, acts as a potent α2 adrenergic agonist, leading to sedation, muscle relaxation, and potential respiratory depression. In this case, a female mistakenly injected herself with xylazine mistaking it for a different medication.

The report discusses naloxone's role beyond opioid overdose, especially regarding substances causing central nervous system (CNS) depression via mechanisms similar to those of opioids. While naloxone is traditionally associated with opioid receptor antagonism, its successful application here suggests potential benefits against non-opioid substances such as xylazine. The report emphasizes the need for higher naloxone dosages than those used for opioid toxicity and suggests more research into its use for treating xylazine intoxication, reflecting on the growing trend of xylazine as a recreational drug adulterant and the resulting health risks.

## Introduction

This report investigates a rare presentation of an isolated xylazine overdose in a human patient and the novel application of naloxone for treatment. Xylazine, a potent α2 adrenergic agonist, mimics the action of norepinephrine by binding to presynaptic α2 receptors in the central nervous system (CNS), thereby inhibiting the release of norepinephrine and dopamine. This inhibition leads to sedative and muscle relaxant effects.

Xylazine intoxication may result from the adulteration of other drugs or from intentional or unintentional ingestion or the administration of xylazine itself. Recent attention has been given to xylazine-associated skin necrosis, although the more significant problem remains its association with opioid-related fatalities. It is not currently clear whether naloxone in standard doses reverses xylazine toxicity in humans [1].

Xylazine's sedative and analgesic properties are thought to arise primarily from its agonistic action on α2 receptors, predominantly located in the brainstem and spinal cord. This activation induces a decrease in sympathetic outflow, resulting in bradycardia, hypotension, sedation, and respiratory depression, which may be fatal. However, possible other pathways including cholinergic, serotonergic, dopaminergic, alpha-1 adrenergic, histaminergic, and opiate mechanisms have been proposed [2].

## Case presentation

An adult female patient with a known history of Hashimoto's thyroiditis and substance use presented at the emergency department exhibiting signs of lethargy. This condition ensued following her self-administration of an intramuscular injection composed of 2 cc of xylazine at a concentration of 100 mg/mL, a dose administered approximately two hours prior to her arrival at the facility. The patient brought the vial of equine tranquilizer from which she had injected herself, and both the patient and her husband denied any sedation or similar symptoms prior to the injection of the xylazine or any additional substance use. The patient was under the mistaken impression that the substance was a steroid meant to manage her thyroiditis and proceeded to inject the equine tranquilizer into her left buttock.

Upon initial evaluation, the patient was notably difficult to arouse. Vital signs included blood pressure of 153/103, heart rate of 71, respiratory rate of 14, and temperature of 97.8°F. The administration of a sternal rub led to a modest improvement in her level of consciousness, as reflected by a Glasgow Coma Scale (GCS) score of 9 (E-2, V-2, M-5). Despite her altered mental state, the patient maintained her ability to protect her airway. Consequently, she was swiftly relocated to a resuscitation room for closer monitoring.

In collaboration with the regional poison control center, a plan was formulated to maintain observation and provide symptomatic management without any immediate interventions discussed. Based on xylazine's mechanism of action and experience with naloxone for clonidine overdoses, the administration of naloxone 2 mg IV was performed to counteract the depressive effects of the tranquilizer. Almost immediately after the administration of naloxone, the patient experienced a notable improvement in alertness and reported a subjective feeling of well-being, with a post-administration GCS score of 15. The patient's toxicologic evaluation including a urinary drug screen was found to be negative for opioids and only tested positive for amphetamines.

Within approximately 30 minutes, the patient again had a recurrence of her decreased level of consciousness. Naloxone was re-dosed, and a continuous infusion was initiated at 2 mg/hour IV. Over the course of this treatment, the patient's mental state progressively improved, though she continued to experience mild lethargy. By the completion of the naloxone infusion in the Medical Intensive Care Unit (MICU) approximately eight hours later, she was noted to have a significant enhancement in both her mentation and alertness levels.

The patient was admitted to the Medical Intensive Care Unit (MICU) for ongoing care and monitoring where she was discharged the next day without any apparent lasting effects.

## Discussion

Naloxone functions as a competitive antagonist at opioid receptors, primarily mu receptors, where it displaces opioids and reverses their effects. It is commonly used in opioid overdoses to restore respiratory function and consciousness. It has long been recognized as a potentially useful adjunct in clonidine overdose where it may influence the regulatory feedback loops that control neurotransmitter release [3].

Clonidine shares a similar mechanism of action with xylazine as an α2 adrenergic agonist. In overdose, the excessive stimulation of these receptors leads to profound CNS depression. While naloxone's antagonistic action on opioid receptors is well-characterized, its role in reversing the CNS depression caused by non-opioid substances such as clonidine or xylazine is not fully elucidated. Some evidence suggests that naloxone may exert effects beyond opioid receptor antagonism, possibly involving interactions within the central adrenergic system that counteract the excessive α2 receptor-mediated CNS depression [4].

Xylazine has been increasingly used as an adulterant in recreational drugs, which may lead to a rise in fatalities among drug users due to its harmful effects, especially when mixed with other drugs of abuse. A 2014 review of 43 cases showed a nearly equal split between non-fatal and fatal outcomes with the use of xylazine, with most non-fatal cases requiring medical intervention [4]. Therapy is typically supportive, including vasopressor support and endotracheal intubation and mechanical ventilation [5].

A recent case series on accidental xylazine intoxication in humans highlights that there is no specific antidote available and questions the efficacy of naloxone in such cases. The case series includes two incidents of accidental human injection with xylazine; in one case, naloxone 1.2 mg IV was administered but did not appear to reverse the toxic effects, while the other case was treated with atropine and vasopressors [6]. Research on clonidine shows that doses much larger than those used to reverse opioid toxicity (up to 10 mg IV) may be required for improvement in mental status [7], which may indicate that an apparent lack of effect of naloxone on xylazine is related to dosing rather than efficacy. The treatment remains largely supportive, focusing on ventilation and hemodynamics, and physicians are cautioned to be vigilant of potential xylazine intoxication in relevant environments.

## Conclusions

In our case, naloxone was utilized emergently due to the patient's deteriorating mental status following xylazine overdose. The rationale for using naloxone was its established efficacy in reversing the CNS depressive effects of clonidine, a drug with a similar mechanism of action to xylazine. Following naloxone administration, the patient exhibited marked improvement in alertness and respiratory function, supporting the hypothesis that naloxone may have utility in reversing the effects of xylazine toxicity. Until further investigation conclusively delineates the role of naloxone in xylazine toxicity, clinicians should consider the use of higher doses of naloxone to avoid intubation if no contraindications exist.
